# Progress on deuterated potassium dihydrogen phosphate (DKDP) crystals for high power laser system application

**DOI:** 10.1038/s41377-022-00929-y

**Published:** 2022-07-29

**Authors:** Mingxia Xu, Baoan Liu, Lisong Zhang, Hongkai Ren, Qingtian Gu, Xun Sun, Shenglai Wang, Xinguang Xu

**Affiliations:** State Key Laboratory of Crystal Materials, Shandong University, Jinan, 250100 China

**Keywords:** Optical materials and structures, Nonlinear optics

## Abstract

In this review, we introduce the progress in the growth of large-aperture DKDP crystals and some aspects of crystal quality including determination of deuterium content, homogeneity of deuterium distribution, residual strains, nonlinear absorption, and laser-induced damage resistance for its application in high power laser system. Large-aperture high-quality DKDP crystal with deuteration level of 70% has been successfully grown by the traditional method, which can fabricate the large single-crystal optics with the size exceeding 400 mm. Neutron diffraction technique is an efficient method to research the deuterium content and 3D residual strains in single crystals. More efforts have been paid in the processes of purity of raw materials, continuous filtration technology, thermal annealing and laser conditioning for increasing the laser-induced damage threshold (LIDT) and these processes enable the currently grown crystals to meet the specifications of the laser system for inertial confinement fusion (ICF), although the laser damage mechanism and laser conditioning mechanism are still not well understood. The advancements on growth of large-aperture high-quality DKDP crystal would support the development of ICF in China.

## Abstract (web summary)

Large-aperture high-quality DKDP crystal with deuteration level of 70% has been successfully grown, which can fabricate the large single-crystal optics with the size exceeding 400 mm for ICF application.

## Graphical abstract

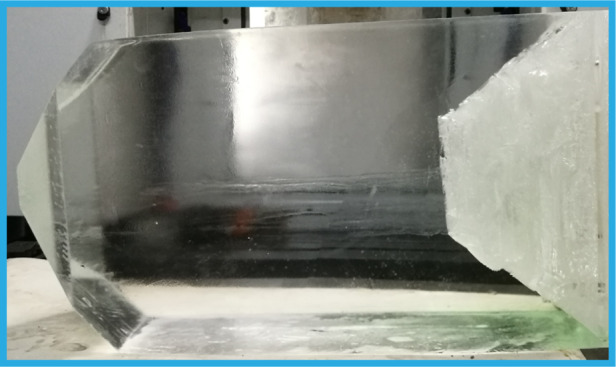

## Introduction

Inertial confinement fusion has attracted the worldwide attention as one of the most promising means to obtain clean energy in the future, which has practical application prospects in solving the problem of energy shortage. In 2021, the National Ignition Facility (NIF) at Lawrence Livermore National Laboratory (LLNL) has made a major step forward, which has finally achieved 70% conversion efficiency, nearly reaching ignition^1^. NIF’s success gives relevant researchers more encouragement. In the past, the research on the growth and performance of large crystal optics has been one of the challenges for the successful construction and operation of NIF^2–5^. Large-aperture potassium dihydrogen phosphate (KDP) and its deuterated analog DKDP are applied as electro-optic switches and frequency conversion crystals in ICF, which are attributed to their excellent performance including wide transmittance, high laser damage threshold, large nonlinear optical coefficient, the feature of growing to large sizes and good processability^2–4,6^. In contrast with KDP crystal, DKDP crystal is commonly used for third harmonic generation (THG) due to its weak transverse stimulated Raman scattering (TSRS), which can efficiently reduce the probability of crystal damage in high power laser systems^7,8^.

So far, laser-induced damage (LID) is still a challenging problem for THG DKDP crystals in the development of high power laser systems, which severely restricts the energy fluence of the output laser and the useful life of crystals. Laser-induced damage of crystal is a rather complex process, which is determined by laser parameters and crystal performance. The effect of laser parameters on LID has been widely investigated including pulse duration, size of the beam spot, laser wavelength^9–12^. From the perspective of the material, LID is related to intrinsic and extrinsic factors. Intrinsic processes include linear absorption, nonlinear effects such as self-focusing, stimulated scattering, collisional (electron avalanche), and multiphoton absorption^13–15^, while extrinsic mechanism could be thermal effects caused by microstructural defects and absorbing inclusions in the materials^13,16,17^. The understanding of laser-induced damage in DKDP crystal helps to improve the crystal quality in the processes of growth and fabrication, which have been proved by the improvement on laser-induced damage thresholds (LIDT) over the past years. However, the mechanism of LID for THG DKDP crystals is not entirely understood yet and the LIDT of DKDP crystals should be further improved with the development of high power laser systems.

In this review, we present the progress of our research on the growth of large-aperture DKDP crystals and some aspects of crystal properties related to its application in high-power laser systems during the past ten years. These works include determination of deuterium content, homogeneity of deuterium distribution, three-dimensional (3D) residual strains, nonlinear absorption, and laser-induced damage resistance. We hope these data can be used for further improvement of the quality of the large-aperture DKDP crystals to meet the stringent requirements of the ICF project. Finally, we propose several application prospects for the DKDP crystals.

## Crystal growth

**Fig. 1:**
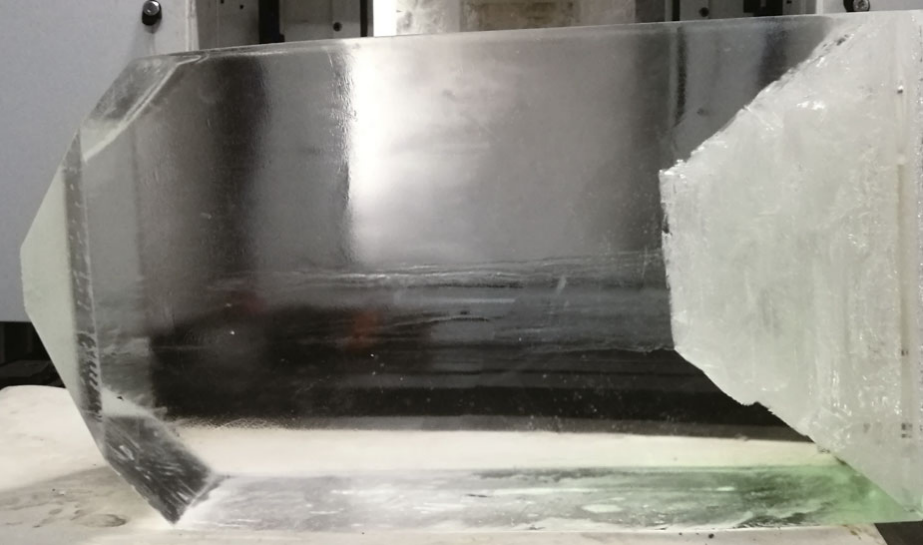
Photograph of large-size DKDP crystal grown by the traditional method

KDP type crystals are usually grown by the aqueous solution method, which mainly includes traditional temperature-reduction method^2^, solution circulating method^18^, rapid growth method^19^. The traditional temperature-reduction method has been widely used to grow large KDP/DKDP crystals, which requires a long growth period of more than two years with a slow growth rate (1–2 mm day^−1^). This will bring high risk and high cost to large-size crystal growth. However, it is likely to obtain high-quality large-size crystals by the traditional method, which can meet the more stringent requirements of quality and size for nonlinear optical crystals, especially for the third harmonic generation (THG) crystal with further development of the ICF engineering. The optimal growth process is determined by studying the DKDP crystal properties under different growth conditions including raw material, growth method, seed orientation, deuterium content^20–23^. Then large DKDP crystal has been successfully grown by traditional method on these bases (Fig. 1), which can fabricate the THG single-crystal optics with the size exceeding 400 mm.

Rapid growth technology has become a hot research topic since the 1990s, which can greatly increase the crystal growth rate (up to 50 mm day^−1^)^24^. Compared to the traditional method, the rapid growth method using a point seed can greatly reduce the volume of regenerated seed caps (the opaque region on the seed crystal) and highly improve the crystal utilization rate. The key problem of realizing the rapid rate of crystal growth is the stability and high supersaturation of an aqueous solution. If supersaturation is not properly controlled, the crystallization will occur not only on the crystal surfaces but also on the invisible small crystal nucleus in the solution, which will cause an undesirable spontaneous crystal in the crystallizer and then lead to the failure of rapid growth for large crystals. Nowadays, rapid growth technology has been successfully developed to grow large-aperture high-quality KDP crystals which can meet the requirements for the fabrication of the optics needed for the ICF^24–29^.

At the same time, the point-seed rapid growth technology has been used to grow DKDP crystals. In 1999, N. Zaitseva et al. designed a continuous filtration system for rapid crystal growth which was used to obtain KDP and DKDP crystals with sizes up to 55 cm^30^. Zhang et al. successfully used point-seed rapid growth technology to grow DKDP crystals with a deuterium content of 98%^31,32^. In 2019, Cai et al. reported that a highly deuterated DKDP crystal with sizes up to 318 mm × 312 mm × 265 mm was grown by the rapid-growth method^33^. This demonstrates that growth by the rapid growth method not only shortens the growth period but also avoids the disturbance of monoclinic crystals, especially for the growth of highly deuterated DKDP crystals. However, the pyramid-prism interfaces in the rapidly grown crystals not only decrease the optical properties of crystals but also lead to obvious phase jump, which will cause the intensity modulation of the propagation beam, especially for the third harmonic beam modulation^34–38^. Researchers have changed the initial seed orientation under the rapid growth conditions to achieve a high yield of THG optics and eliminate the pyramid-prism interfaces^4,39–41^.

For tripler DKDP crystals grown by rapid-growth technology, although successfully producing the large-size optics meeting NIF requirements at LLNL, the material quality did not completely meet all NIF specifications. Therefore, all the tripler DKDP crystals used in NIF were grown by the traditional method^5^. Chen et al. successfully used the rapid growth method to grow a cuboid DKDP crystal without a pyramidal sector^41^. Whether the resulting material grown by this method meets the requirements of the project needs to be further verified.

## Crystal characterization

### Determination of deuterium content in DKDP crystal

Deuterium content is a very important parameter for DKDP crystal, which is defined as the molar percentage of deuterium in the total number of hydrogen atoms in the crystal lattice. The chemical and physical properties of DKDP crystals are highly sensitive to the deuteration level of DKDP crystals, such as lattice parameters, phase-transition temperature, refractive index, optical properties^16,42–44^. Therefore, an accurate measurement of the degree of deuteration in DKDP crystal is extremely important for its applications in high-power laser systems.

G. M. Loiacono et al. reported the variation in the ferroelectric transition temperature with deuteration in 1974, which was almost dependent linearly on its deuteration level^45^. Yaksin et al. presented that Raman scattering spectra could be used to measure the deuteration degree of DKDP crystals^46^. Huser et al. demonstrated that the Raman shift of the main PO_4_ vibration peak varied linearly with the deuteration level^47^. Li et al. proposed a new method to measure the deuterium content of DKDP crystals, which mainly used thermo-gravimetric apparatus to weigh the initial DKDP crystal sample and products of thermal decomposition^48^. Liu et al. found that there was a linear relationship between refractive index and deuteration degree of DKDP crystal, which might become a potential method to determine the deuterium content^49^. However, all of the above methods measured the variation of the physical and chemical properties of crystals with the deuterium content to determine the deuterium content of DKDP crystals indirectly.

**Fig. 2:**
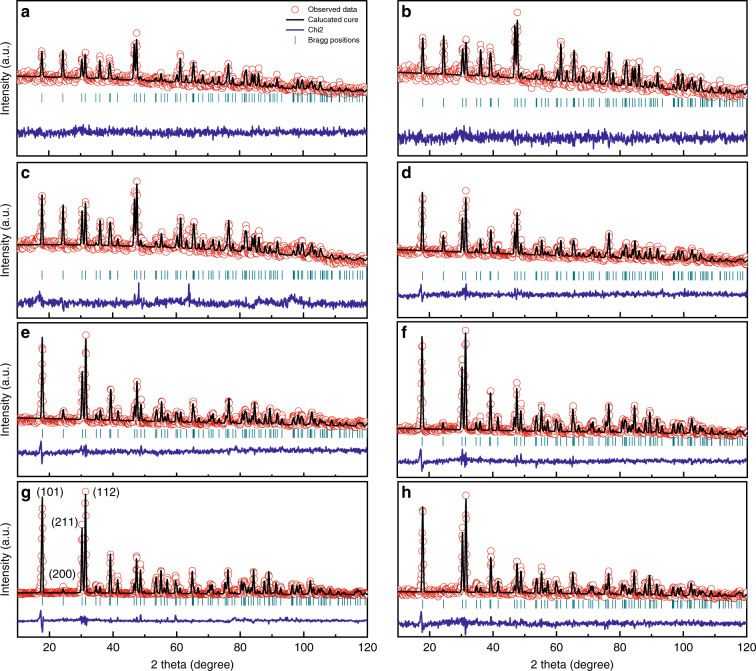
Neutron diffraction patterns of DKDP crystals grown with different solution deuteration levels (D_s_): **a** 0%, **b** 40%, **c** 55%, **d** 65%, **e** 80%, **f** 92%, **g** 98%, and **h** 99.5%^51^. Reprinted with permission from^51^ Copyright, The Optical Society

The neutron scattering length of the deuterium atom is larger than that of other atoms in DKDP crystal, so neutron diffraction can be used as an effective technique to directly determine the deuterium content of DKDP crystals^50,51^. Neutron powder diffraction data were measured by the high-pressure neutron powder diffractometer (Fig. 2). Table 1 showed the deuterium content of DKDP crystals (D_c_) grown from different solution deuteration levels (D_s_), which could be obtained from the refined neutron diffraction data.

**Table 1:** The deuterium contents of DKDP crystals and their growth solutions^51^

Ds(%)	0	40.0	55.0	65.0	80.0	92	98.0	99.5
Dc(%)	0	29.1	45.1	55.1	73.8	83.9	93.8	99.1

Reprinted with permission from^51^ Copyright, The Optical Society

**Fig. 3:**
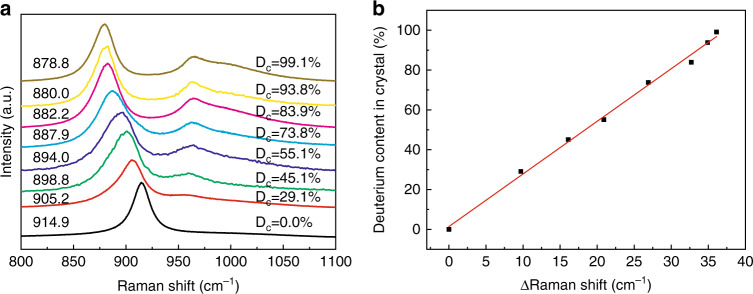
**a** Raman spectra of DKDP crystals^51^ and **b** dependence of the variation in Raman shift of the totally symmetric PO_4_ vibration in DKDP on the degree of deuteration^51^. Images reprinted with the following permission: (**a**)^51^, (**b**)^51^ from The Optical Society

Then the results of neutron diffraction were used to calibrate the relationship between the deuterium content and the variation of PO_4_ vibration peak in Raman spectra and the absorption bands in IR spectra, respectively. According to the Raman shifts of PO_4_ vibration peak of KDP and DKDP crystals with different degrees of deuteration (Fig. 3a), the relative Raman shift [Δν_1_ = ν_1_(KDP) − ν_1_(DKDP)] was used to determine the deuterium content of DKDP crystals. There was a linear relationship between the relative Raman shift and D_c_ (Fig. 3b), as follows:

1 $${{{\mathrm{D}}}}_{{{\mathrm{c}}}}\left( \% \right) = 2.64\left[ {\% \left( {{{{\mathrm{cm}}}}^{ - 1}} \right)^{ - 1}} \right] \times \left[ {\nu _1({{{\mathrm{KDP}}}}) - \nu _1({{{\mathrm{DKDP}}}})} \right]$$

where ν_1_(KDP) and ν_1_(DKDP) were the Raman shifts of the PO_4_ vibration peak (cm^−1^) for KDP and DKDP crystals, respectively.

**Fig. 4:**
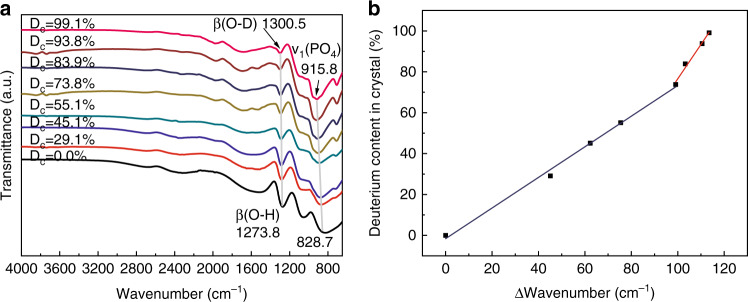
**a** IR spectra of DKDP crystals^51^ and **b** dependence of the variation in IR [β(DKDP)-β(KDP) + ν_1_(DKDP)- ν_1_(KDP)] of the total β(O-H/D) and ν_1_(PO_4_) vibration in DKDP on the degree of deuteration^51^. Images reprinted with the following permission: (**a**)^51^, (**b**)^51^ from The Optical Society

Similarly, the dependence of the variation in β(O-H/D) (it presents stretching vibration of the O-H or O-D bond) and ν_1_(PO_4_) of the IR spectra on the deuterium content was showed two different kinds of a linear relationship between them (Fig. 4). When the degree of deuteration was less than 73.8%, the linear relationship was shown as follows:

2 $${{{\mathrm{D}}}}_{{{\mathrm{c}}}}\left( \% \right) = 0.75\left[ {\% \left( {{{{\mathrm{cm}}}}^{ - 1}} \right)^{ - 1}} \right] \times \left\{ {\left[ {\beta \left( {{{{\mathrm{DKDP}}}}} \right) - \beta \left( {{{{\mathrm{KDP}}}}} \right)} \right] + \left[ {\nu _1\left( {{{{\mathrm{DKDP}}}}} \right) - \nu _1\left( {{{{\mathrm{KDP}}}}} \right)} \right]} \right\}$$

where β(KDP) and β(DKDP) were the β(O-H/D) absorption bands of KDP and DKDP crystals, respectively. When the degree of deuteration was more than 73.8%, the relationship could become the following Eq.:

3 $${{{\mathrm{D}}}}_{{{\mathrm{c}}}}\left( \% \right) = 1.681\left[ {\% \left( {{{{\mathrm{cm}}}}^{ - {{{\mathrm{1}}}}}} \right)^{ - 1}} \right] \times \left\{ {\left[ {\beta \left( {{{{\mathrm{DKDP}}}}} \right) - \beta \left( {{{{\mathrm{KDP}}}}} \right)} \right] + \left[ {\nu _{{{\mathrm{1}}}}\left( {{{{\mathrm{DKDP}}}}} \right) - \nu _1\left( {{{{\mathrm{KDP}}}}} \right)} \right]} \right\} - 91.5$$

Both spectral techniques could be applied to determine the deuterium content of DKDP crystals with solution deuteration level less than 92%, while IR spectroscopy should be more suitable to measure more highly deuterated DKDP crystals.

### Homogeneity of deuterium distribution in DKDP crystal

**Fig. 5:**
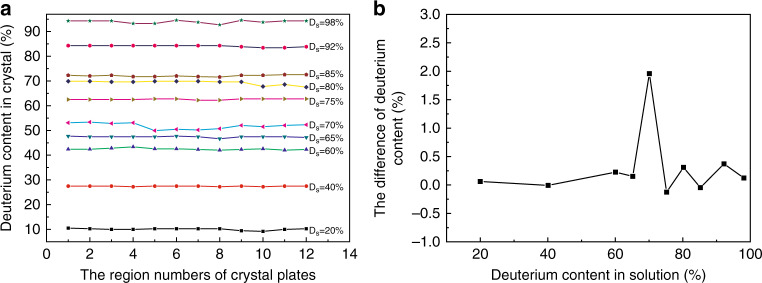
**a** Deuterium distribution in DKDP crystals grown from solutions with different deuterium contents and supersaturation levels ranging from 0.04 to 0.06^52^ and **b** difference in the average deuteration levels between the pyramidal and prismatic sections (their size represent the error bars)^52^. Images reproduced with the following permission: (**a**)^52^, (**b**)^52^ from Wiley-VCH

**Fig. 6:**
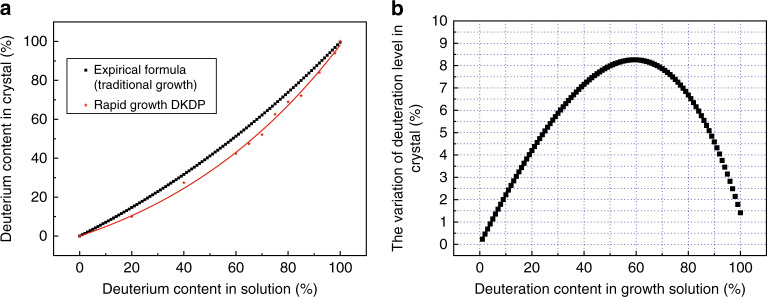
**a** The dependence of average deuterated-level in crystals grown by rapid growth with supersaturation of 0.04 to 0.06 on the deuteration level in solution and empirical formula used to traditional growth crystals^52^ and **b** difference in the segregation coefficients of the deuteration level between traditional and rapid growths (average)^52^. Images reproduced with the following permission: (**a**)^52^, (**b**)^52^ from Wiley-VCH

**Fig. 7:**
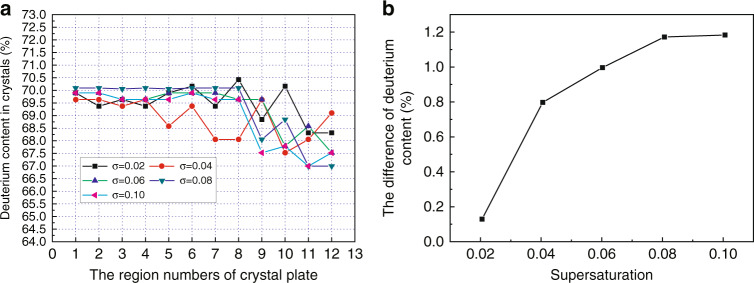
**a** Deuterium distribution in DKDP crystals grown from 80% deuterated solutions at different supersaturation levels^52^ and **b** the difference in the average deuterium content between the pyramidal and prismatic sections^52^. Images reproduced with the following permission: (**a**)^52^, (**b**)^52^ from Wiley-VCH

It is well-known that the homogeneity of deuterium distribution in DKDP crystals is very important for optical applications. In the growth process of DKDP crystals, the inhomogeneity of deuterium distribution is closely related to the variation of growth parameters. For a rapidly grown DKDP crystal with the size of 65 mm × 65 mm × 113 mm, the homogeneity of deuterium distribution was measured by the Raman spectra and the results showed the maximum discrepancy of deuterium content was 5.4%^36^. The effect of supersaturation on deuteration distribution in DKDP crystal was further investigated by Raman and infrared spectroscopy^52^. The deuterium distributions in DKDP samples with different deuteration levels were obtained (Fig. 5a) and then the homogeneities between pyramidal and prismatic sections were compared (Fig. 5b). The maximum discrepancy of the average deuterium content between the two sections was approximately 2% when the solution deuteration level is 70%, while that of others was less than 1%. The deuteration segregation coefficient of rapid-growth DKDP crystal with high supersaturation was smaller than that of traditional-growth crystal with low supersaturation in solution with the same deuterium content (Fig. 6). For the rapid-growth DKDP crystal grown with different supersaturation, deuterium distributions in crystals were shown in Fig. 7. The results indicated that the deuterium content in the pyramidal section suffered only minimal disruption from variations in supersaturation, while that in the prismatic section presented a large fluctuation (Fig. 7a). The difference in the average deuterium content between the two sections increased with increasing supersaturation, which indicated serious inhomogeneity of deuterium distribution in the whole crystal (Fig. 7b).

The deuterium homogeneities of large-size DKDP crystals grown by traditional growth method and rapid growth method were investigated in situ by Raman spectroscopy, respectively^37,53^. The results indicated that the inhomogeneity of deuterium content was estimated to be 0.12% for the traditional-growth DKDP crystal, which meant its influence on the third harmonic generation (THG) efficiency could be neglected. However, the deuterium gradient was about 0.2% cm^−1^ for the rapid-growth DKDP crystal, which will lead to about a 5% reduction of the THG efficiency at 3 GW cm^−2^ of fundamental radiation^37^.

### Residual strain and stress in single crystal

In the growing and machining process of DKDP crystals, residual stress can be introduced in the crystal by the lattice deformation, which may be caused by some unavoidable changes such as the H/D isotopic exchange, temperature variation, defect generation, introduction of external forces^50,54–56^. The residual stresses not only affect the mechanical properties of the crystal but also affect the other properties. Thus, it is very important to investigate the residual stress in DKDP crystal for its applications in high power laser systems.

The residual stress in a single crystal is characterized mainly by the photoelastic method, X-ray technique, and neutron diffraction technique. Compared with the other two methods, the neutron diffraction technique has obvious advantages for its deep penetrability through most of the materials without damage occurring, which has been maturely applied to the research of stress and strains in polycrystal materials^57–59^.

**Fig. 8:**
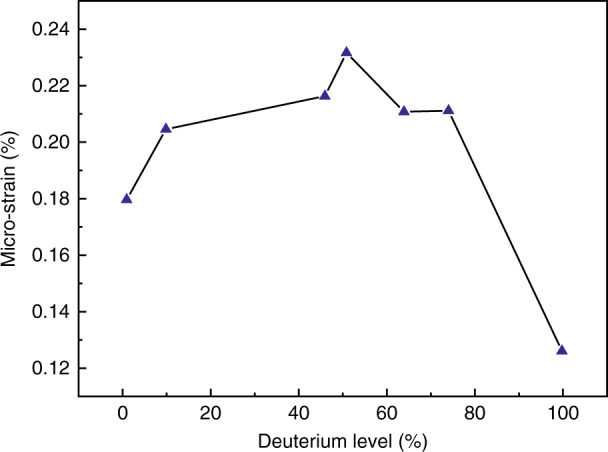
The average value of the micro-strain of {323} each faces of experimental sample^60^. Reproduced with permission^60^ Copyright, Wiley-VCH

DKDP crystals with different deuterium contents were grown by rapid growth method and the three-dimensional (3D) residual strains in single crystals were investigated by neutron diffraction technique^52,60^. The magnitude of residual strains (*ε*_*ij*_) in crystallographic coordinate was 10^−3^ to 10^−4^ and the normal strains along [001] direction were always compressive (Table 2). Then according to the values of macroscopic strains (*ε*_*ij*_) ^60^ and elastic stiffness constant (*C*_*ij*_)^61^, the residual stresses were calculated by Hooke’s law. It was illustrated that the value of shear stress was much smaller than that of normal stress and the magnitude of the macro-strain in DKDP crystals was independent of the deuterium content (Table 3). The average micro-strains of the {323} faces were calculated from the values of full-width at half-maximum for the diffraction peaks (Fig. 8), which indicated that there was a trend of increasing first and then decreasing for the average the micro-strain, with the maximum value occurring at 50% deuterium. In addition, the potential sources of the residual strains and stresses in single crystals were attributed to the defects including dislocations, interstitial defects, and vacancy defects. However, the influence of these defects on the macroscopic stress needs further research.

**Table 2:** The macro-strain ε_ij_ in crystalline coordinate^60^

Dc(%)	Dc	*ε*_*11*_	*ε*_*22*_	*ε*_*33*_	*ε*_*23*_	*ε*_*13*_	*ε*_*12*_
0	0	0.00690(03)	0.00420(08)	−0.00710(20)	0.00080(03)	0.00080(01)	0.00130(03)
9	0.09	0.00180(18)	−0.00110(17)	−0.00100(00)	−0.00010(01)	−0.00110(01)	−0.00140(01)
45	0.45	0.00570(18)	0.00190(16)	−0.00400(03)	0.00030(04)	0.00060(05)	0.00090(04)
50	0.5	0.00220(26)	0.00260(23)	−0.00390(07)	0.00060(02)	−0.00010(04)	0.00030(02)
63	0.63	0.00090(05)	0.00000(07)	−0.00280(08)	−0.00050(01)	−0.00020(01)	0.00030(00)
73	0.73	0.00150(06)	0.00150(06)	−0.00290(04)	0.00040(01)	0.00040(01)	0.00020(02)
99	0.99	−0.00030(19)	0.00160(03)	−0.00210(03)	−0.00010(02)	−0.00010(05)	−0.00010(01)

Reproduced with permission^60^ Copyright, Wiley-VCH

**Table 3:** The macro-stress in crystallographic coordinate (MPa)^60^

x	0	0.09	0.45	0.50	0.63	0.73	0.99
*σ*_*11*_	320.5 ± 3.1	107.5 ± 9.5	294.4 ± 9.7	77.0 ± 13.3	26.9 ± 3.7	52.0 ± 3.8	−50.5 ± 12.3
*σ*_*22*_	136.9 ± 6.4	−89.7 ± 9.4	36.0 ± 8.5	104.2 ± 11.7	−34.3 ± 4.5	52.0 ± 3.5	78.7 ± 2.9
*σ*_*33*_	−244.0 ± 10.9	−43.0 ± 3.6	−124 ± 2.1	−147.0 ± 1.4	−34.3 ± 5.4	−115.0 ± 3.0	−92.0 ± 3.3
*σ*_*23*_	7.8 ± 0.2	−8.4 ± 0.1	5.4 ± 0.3	1.8 ± 0.1	1.8 ± 0.1	1.2 ± 0.1	−0.6 ± 0.1
*σ*_*13*_	9.6 ± 0.4	−13.2 ± 0.1	7.2 ± 0.6	−1.2 ± 0.5	−2.4 ± 0.1	4.8 ± 0.1	−1.2 ± 0.6
*σ*_*12*_	9.6 ± 0.4	−1.2 + 0.1	3.6 ± 0.5	7.2 ± 0.3	−6.0 ± 0.2	4.8 ± 0.1	−1.2 ± 0.2

Reproduced with permission^60^ Copyright, Wiley-VCH

### Nonlinear optical property of DKDP crystal

The absorption of UV light usually increases nonlinearly with the irradiated laser intensity, which has become one of the major problems for the frequency-conversion crystal. A. Melninkaitis et al. found that when the energy density increased from 0.1 J cm^−2^ to 3 J cm^−2^, the absorption of KDP crystal with a length of 1 cm at 355 nm increased 1.9%^62^. The nonlinear absorption (NLA) is usually attributed to multiphoton absorption. For the frequency-conversion crystals, the NLA not only results in the energy loss of the laser beam but also causes potential damage to the crystals^63,64^. At present, the NLA of KDP crystals at different wavelengths have been investigated, while the research for the NLA of DKDP crystals has been few reported^65–68^.

**Fig. 9:**
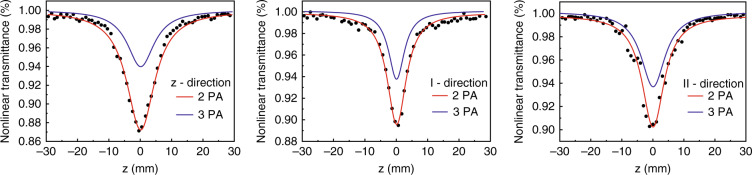
NLA curves of different cutting^69^. Adapted with permission from^69^ Copyright, The Optical Society

**Fig. 10:**
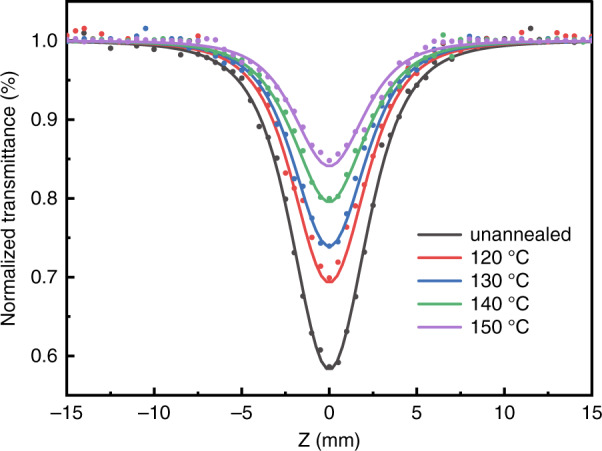
NLA curves of the samples annealed at different temperatures^70^. Reproduced from ref. ^70^ with permission from The Royal Society of Chemistry

**Fig. 11:**
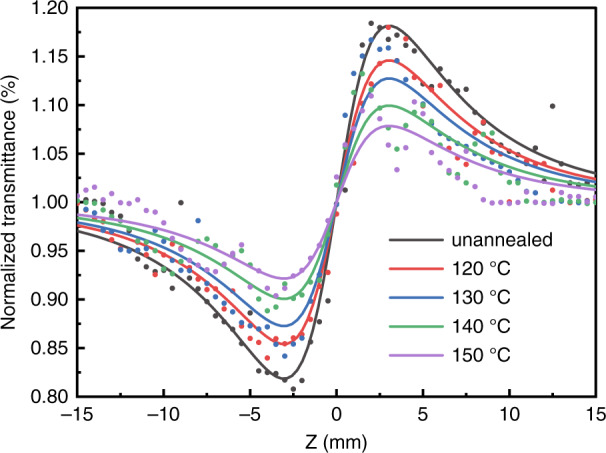
NLR curves of the samples annealed at different temperatures^70^. Reproduced from ref. ^70^ with permission from The Royal Society of Chemistry

The NLA of DKDP crystals with deuterium content of 70% was measured by the Z-scan method using the nanosecond and picosecond Nd: YAG laser with a wavelength of 355 nm, respectively^69,70^. Both results showed that the NLA at 355 nm was assigned to two-photon absorption (Fig. 9). The NLA and nonlinear refraction (NLR) of DKDP crystals were obviously influenced by thermal annealing, which decreased with the increasing annealing temperatures (Figs. 10 and 11). In addition, the laser-induced damage of 70% deuterated DKDP crystal was considered to relate to NLA and NLR, which should be further researched.

## Improvements on laser-induced damage resistance

The most important property of KDP and DKDP crystal used as nonlinear optics in high power laser systems such as NIF or SG III is the laser-induced damage resistance. The mechanism of laser-induced damage for these materials and how to improve their resistance to laser damage have attracted intensive attention since the construction of the large-aperture laser system for the Inertial Confinement Fusion. From the perspective on the microstructure of crystalline materials, many microscopic-scale defects such as impurities, dislocations, inclusions, growth boundaries, etc. generated during the growth of single crystals and their influences on laser induced damage have been studied^71–73^. Along with the improvement in the material manufacturing process, the above-mentioned defects can be well removed or controlled at a very low level, and studies have shown that the damage phenomenon of crystal optics is not substantially related to those defects. From the reported damage behavior and mechanism of crystal optics irradiated by ultraviolet laser with high fluences, the initial bulk damage of crystal is related to the nano-scale cluster defects inside the material, which become the light absorption center when irradiated by high power laser^16,74^. The damage characteristics of materials are closely related to the size and density of absorbing defects. The distribution of such absorbent defects is well below the detection limit of common optical techniques, so it is difficult to obtain complete information such as their chemical species, sizes, and distribution characteristics.

At the beginning of research on DKDP crystal growth, the effect of purity of raw materials and variable crystal growth parameters on the laser-induced damage property have been investigated. Although the thermal annealing method which has been confirmed for effectively improving the optical property of KDP crystal is seldom used for DKDP crystal as its lower phase transition temperature. Improved thermal annealing for DKDP crystal will be introduced here, as well as laser conditioning. Moreover, the connection between the growth parameters and the laser induced damage resistance of DKDP crystals could be revealed from the defects generated during the growth process. Some experiments and theoretical studies on the effect of defects on the properties of KDP/DKDP crystals will be present in the third section.

### Impurities and growth conditions

Normally, there always exist some impurities in the aqueous solution for DKDP crystal growth. The impurities in the solution may come from starting salts, deuterated water, intention dopants, or dissolved materials of the vessel. Even now the nature origin of bulk damage is unclear, or no impurities or defects that are directly related to the formation of pinpoint damage have been detected. The impurities with ion state in the solution could be absorbed on the growing surface and modify the movement of the growth steps, the growth habit, and the crystallinity of DKDP crystals.

**Fig. 12:**
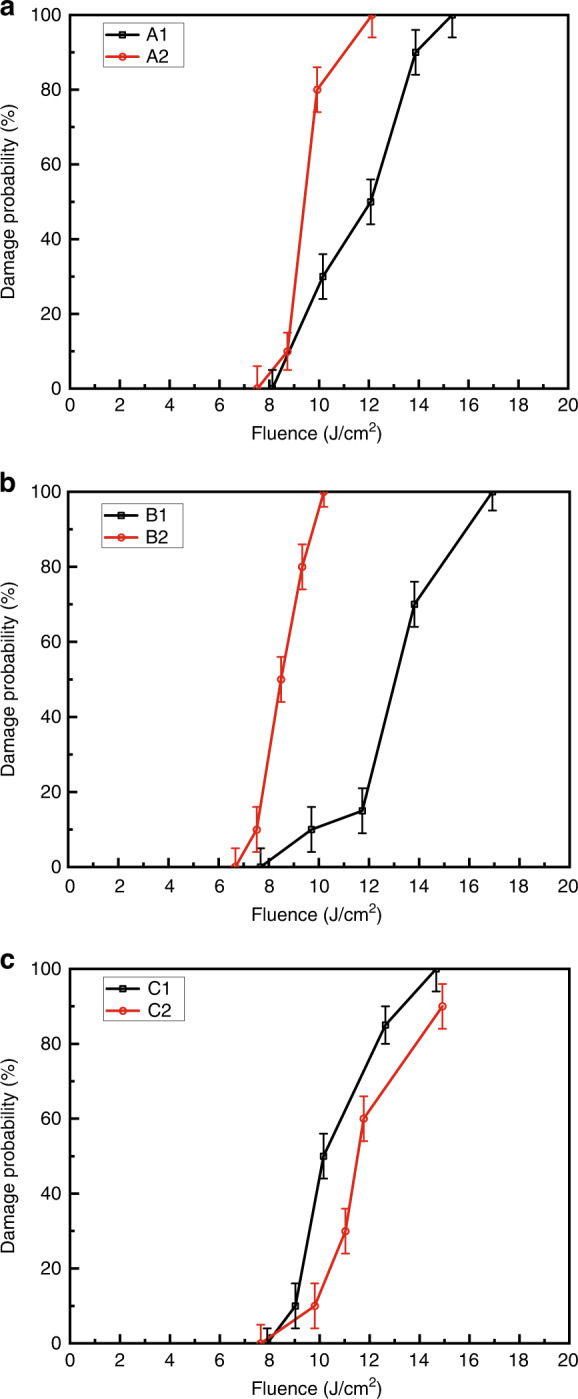
Bulk damage probability curves of DKDP crystal for 355 nm using tripler samples. **a** A1, A2, grown with raw material A^76^, **b** B1, B2, grown with raw material B^76^, and **c** C1, C2, grown with raw material C. 1 means conventional growth, 2 means rapid growth^76^. Images reprinted with the following permission: (**a**)^76^, (**b**)^76^, (**c**)^76^ from Chinese Laser Press

In our previous work, we concentrated on impurities from the starting salt. Sun et al.^75^ have used the conventional temperature-reduction method to grow KD_2_PO_4_ (DKDP) crystals from 85% deuterated solution synthesized by two kinds of KH_2_PO_4_ (KDP) starting salts. The crystals which were grown from material containing higher-level metallic impurity (Fe~10 ppm) have more ultraviolet optical absorption than that grown from material with lower-level impurity (Fe~1 ppm). The crystal grown with high purity material has superior laser damage resistance at 1064 nm, but no significant difference (<15%) at 355 nm compared to the crystal grown with lower purity material. Liu et al.^76^ have used three kinds of KH_2_PO_4_ salts (named as A, B, and C; A and B were purer than C, the mass content of main metal ions were below 1 ppm) to grow DKDP crystals by conventional and rapid growth methods, respectively. The 1-on-1 testing damage probabilities curves of these DKDP crystals are shown in Fig. 12. For conventional growth, the difference in material purity does not lead to a visible variation of damage probabilities between the three crystals A1, B1, and C1. Normally, the rapidly grown crystals have poor laser damage resistance compared with the conventionally grown crystals. However, there will be comparable damage resistance to conventionally grown crystals when the crystal is grown at a lower growth rate (e.g., about 3 mm day^−1^ for crystal C2). With identical other growth parameters, the damage resistance in DKDP is slightly independent of raw material with the mass content of main metallic impurity below 1 ppm.

Burnham et al.^72^ have reported that laser induced pinpoint bulk damage of DKDP crystals at 351 nm depends on growth conditions such as growth temperature, and continuous filtration. Negres et al.^77^ have proved that the damage performance of DKDP crystals was dependent on growth temperature and non-correlated with impurity concentration. In addition, the damage resistance in DKDP is independent of the growth rate at constant growth temperature. In our work, continuous filtration is a key procedure during the process of large-aperture DKDP crystal grown in a 1000 L crystallizer. It was found that the samples from different locations within the boule of conventionally grown DKDP crystal with continuous filtration have a slight difference in laser damage resistance at 355 nm (Table 4). Like the previous report, the sample from the last grown pyramidal section (represent material grown at low temperature) has higher laser damage resistance. Moreover, we have grown four DKDP crystals with 60% deuterated level at different temperatures using the point seed technique but without continuous filtration. The aspect ratio of crystal grown at high temperature is higher than that grown at low temperature. The crystalline perfection and the laser induced damage threshold at 1064 nm decrease with the decrease of growth temperature. DeMange et al.^78,79^ have reported that precursors responsible for damage initiation at 1064 nm are different from those at 355 nm. Thus, there appears a different trend for laser damage resistance at 1064 nm.

**Table 4:** The LIDTs of 70% DKDP crystal from different locations within the boule by 1-on-1 and R-on-1 measurement

Samples	Fluence (J cm^−2^, 5.8 ns, 355 nm)	
	1-on-1	R-on-1
1# (near seed cap)	0.8	2.9
2# (mid prism)	1.9	7.2
3# (late prism)	1.6	5.2
4# (last pyramid)	2.2	7.5

### Thermal annealing and laser conditioning

In addition to using high-purity raw materials and optimizing growth processes, additives, dopants, and post-treatment can also be used to improve the quality of the KDP and DKDP crystals. The post-treatment including thermal annealing and laser conditioning could be used to increase the activation energy of lattice motion, release excess energy, reduce the internal stress of the crystal, and improve the crystallinity. Then the laser damage resistance and optical homogeneity can be improved.

**Fig. 13:**
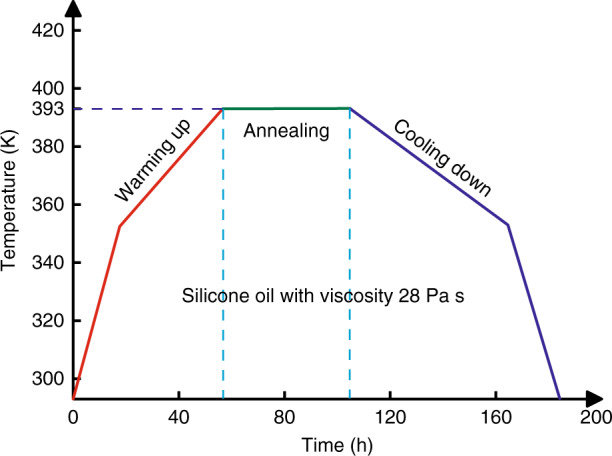
Annealing process for DKDP crystals with a deuterated level of 98%^85^. Reproduced from ref. ^85^ with permission from The Royal Society of Chemistry

**Fig. 14:**
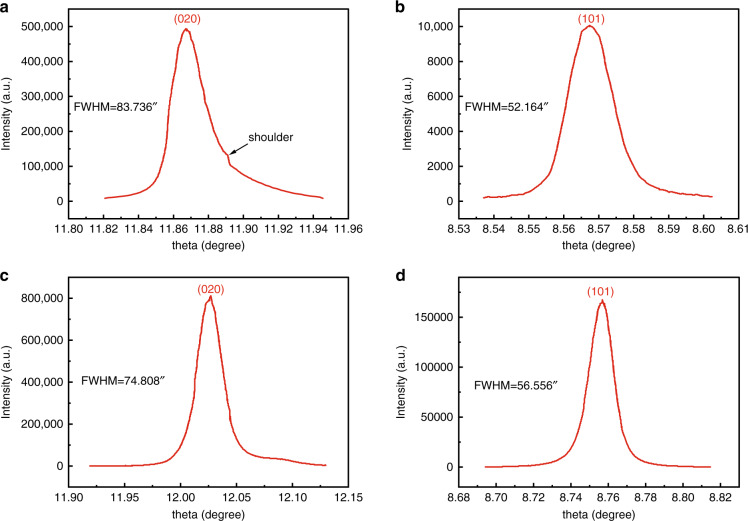
X-ray rocking curves of (020) and (101) slices before and after the annealing process. **a** (020) slice before annealing^85^, **b** (101) slice before annealing^85^, **c** (020) slice after annealing^85^, **d** (101) slice after annealing^85^. Images reproduced with the following permission: (**a**)^85^, (**b**)^85^, (**c**)^85^, (**d**)^85^ from The Royal Society of Chemistry

**Fig. 15:**
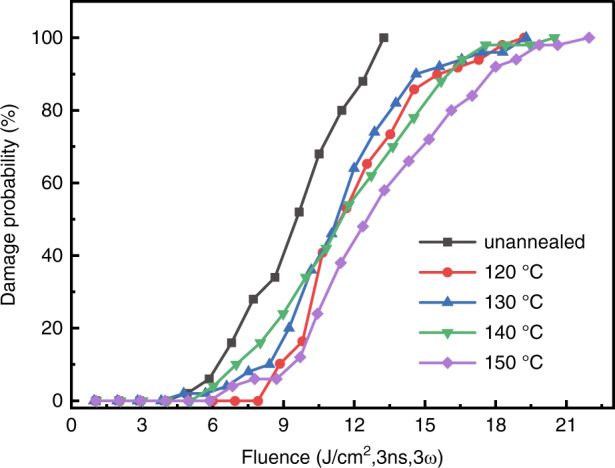
Damage density curves obtained at 3ω, 3 ns for different annealing temperatures^70^. Reproduced from ref. ^70^ with permission from The Royal Society of Chemistry

Early in the 1980s, Swain et al.^80,81^ have investigated the effect of subthreshold irradiation on the 1064 nm bulk laser damage resistance of KDP crystals. The combination of baking (thermal annealing) and laser irradiation with sub-threshold fluence was more effective in improving the bulk damage threshold for all laser pulse duration from 1 to 20 ns. Fujioka et al.^82^ also proved that the thermal conditioning was effective for the rapidly grown crystals to improve the damage threshold (1064 nm, 1 ns, 1- on - 1 test), as well as reducing the strain in the crystal. Guillet et al. have reported that thermal annealing could indeed improve laser damage resistance at 3ω for DKDP crystal, the resistance increment depends on the pulse length^83^. In Fu’s work^84^, the optical homogeneity of the samples for KDP and DKDP have been improved after thermal conditioning, while the laser damage threshold and light absorption coefficient showed no significant change. Sun et al. also confirmed that thermal conditioning was an effective method to improve the transmittance in ultraviolet wavelength and no improvement on laser damage thresholds for both 1ω and 3ω^75^. The thermal annealing temperature is usually below 363 K for DKDP crystals, while the annealing temperature of KDP crystals is about 423 K. The relative low annealing temperature leads to no significant improvement of thermal annealing on the laser damage resistance for DKDP crystals. Thus, we investigated in detail the high temperature phase transition of KD_2_PO_4_ crystal. And a new annealing method was developed by using silicone oil as a protective ambient environment under higher temperatures, the hydrogen-deuterium exchange can be inhibited under this environment^85^. After the annealing process (Fig. 13), the crystallinity of the DKDP crystal is improved (Fig. 14). Moreover, the improvement on 3ω laser damage resistance after thermal annealing was confirmed by Cai et al. (Fig. 15)^70^. This new thermal annealing method has been set as an important procedure in our production of large-aperture DKDP optics.

Since laser conditioning by pre-exposure to subthreshold laser pulse have been proved as an effective method to improve the laser damage resistance for KDP and DKDP crystals. Protocols for laser conditioning on optics used in large-aperture laser systems gain intensive attention from researchers, as well as the characteristics and mechanisms of laser conditioning for KDP/DKDP crystals^11,86–90^. Zhao and Hu have studied the absorption property and mitigation of scattering defects for DKDP crystals after laser conditioning^91–93^. We have measured the laser induced damage threshold of many DKDP crystals, one typical result is given in Table 5. It can be seen that the LIDT of R-on-1 measurement which has the effect of laser conditioning is obviously higher than that of 1-on-1 measurement. The efficiencies of laser conditioning are dependent on the growth process of DKDP crystals. Liao et al. developed an Absorption Distribution Model (ADM) to map the defect population variability in DKDP crystals^90^. It’s necessary to use this kind of model to optimize the laser conditioning protocol for crystals with different damage qualities.

**Table 5:** Laser induced damage thresholds of tripler samples for DKDP crystals

Raw materials	0%LIDT (J cm^−2^) (355 nm, 8 ns)			0%LIDT (J cm^−2^) (355 nm, 8 ns)		
	Conventional growth			Rapid growth		
	1-on-1	R-on-1	Gain	1-on-1	R-on-1	Gain
A	8.5	16.9	2.0X	8.5	13.9	1.6X
B	9.6	13.9	1.4X	7.2	11.1	1.5X
C	8.1	15.8	2.0X	8.8	12.8	1.5X

### Defects for damage initiation in KDP/DKDP crystal

In the past decades, many efforts including experimental measurements or theoretical studies have been devoted to exploring the defects in KDP/DKDP crystals which are thought to be relative to the laser damage initiation^94–103^. The observed results based on microscopic techniques such as laser scatter diagnostic and fluorescence microscopy has indicated that the scattering defects and fluorescent clusters didn’t strongly correlate to the location of bulk damage sites^94,98^. The experimental work using optical absorption and electron paramagnetic resonance spectroscopies has identified four main intrinsic (atomic) defects in KDP crystals, such as [HPO_4_]^−^ center, [HPO_4_]^0^ center, (H)^0^ atoms, and [PO_3_]^2−^ center^95–97^. S. G. Demos et al.^16^ developed a novel experimental approach to probe the electronic structure of damage precursors. In their experiment, the sample was irradiated to spatially and temporally overlapping 2ω and 3ω pulses at various fluence combinations and the density of pinpoint damage sites as a function of the ratio of the 3ω effective fluence over the corresponding 2ω fluence was estimated. The results could be reproduced by using a multi-level electronic structure model. Based on the experimental observations and the modeling results, they proposed that clusters of holes trapped near oxygen sites are the constituent defects of the damage precursors. However, it remains a challenge to directly detect this kind of defect in experiment.

**Fig. 16:**
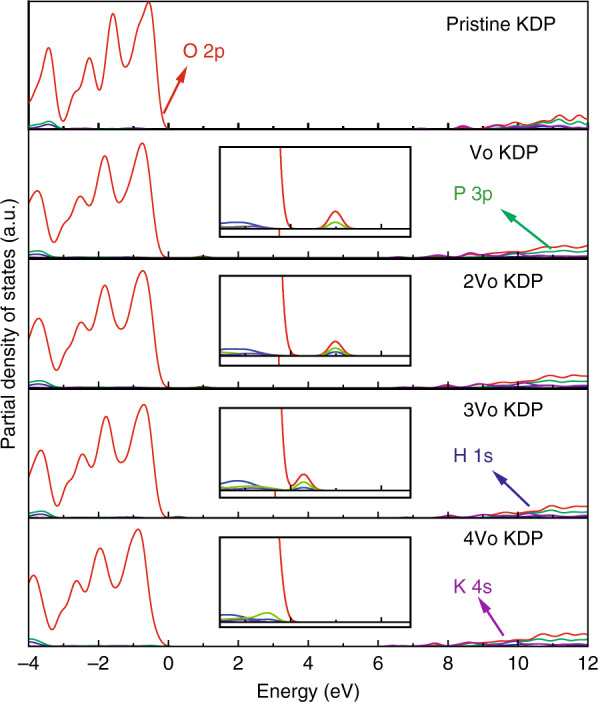
The partial density of states (PDOS) for the oxygen vacancy cluster defects with different concentration^108^. Reproduced from ref. ^108^ with permission from The Royal Society of Chemistry

**Fig. 17:**
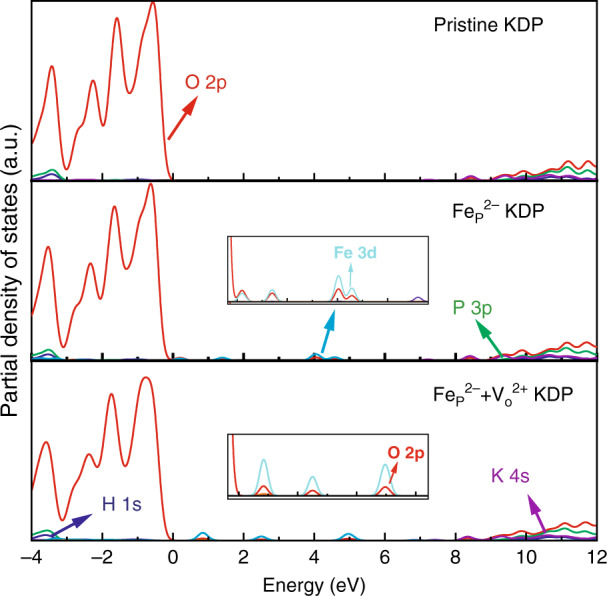
The partial density of states (PDOS) for Fe_p_^2−^ + V_o_^2+^ defect and Fe_p_^2−^ + V_o_^2+^ cluster defect for the KDP crystal^108^. Reproduced from ref. ^108^ with permission from The Royal Society of Chemistry

On the other hand, theoretical studies by density functional theory have been carried out to investigate the electronic structure of point defects in KDP crystal. The modeling results such as band-gap narrowing or a new state in the band-gap can explain the variation of optical absorption property for this material. In our recent works, the first-principle method was applied to investigate the intrinsic, interstitial, and metal ion defects in KDP and ammonium dihydrogen phosphate (ADP) crystals^104–108^. However, crystal defects often do not exist singly but combine to form clusters. Thus, Sui et al. have investigated the structure, stability, and electronic structures of the oxygen vacancy cluster defect and Fe_p_^2−^ + V_o_^2+^ cluster defect for the KDP crystal. The partial density of states (PDOS) of the oxygen vacancy and cluster defects are shown in Figs. 16 and 17^108^. The concentration of oxygen vacancy defects has little effect on the electronic structure, only leading to the torsion of the surrounding bonds. The defect state induced by Fe_p_^2−^ defect could introduce three defect states at 0.5, 1.2, and 4.0 eV, respectively. When the Fe_p_^2−^ + V_o_^2+^ cluster defect forms, these defect states shift about 1.0 eV close to conduction band minimum (CBM). This variation would influence the transient optical absorption under irradiation by a high-intensity laser pulse. As the most common defects in KDP/DKDP crystals, we also studied the effect of dislocation by theoretical modeling. The structure, total system energies, electronic structures, and optical absorption of the [010] and [011] screw dislocations in KDP crystals have been investigated using the density functional theory with Perdew-Burke-Ernzerhof and Heyd-Suseria-Ernzerhof (HSE06) functionals^109,110^. The results show that these dislocations would contribute to a large nonlinear absorption, enhance the crystal to absorb more laser energy, and decrease the laser-induced damage threshold of the KDP crystal.

## Conclusios and perspectives

Large-aperture high-quality DKDP crystals have been successfully grown by the traditional method, which can fabricate the large single-crystal optics with size exceeding 400 mm. Raman spectroscopy as a non-destructive detection method is applied to determine the deuterium content and its distribution in DKDP crystal, which has been calibrated by neutron diffraction. The three-dimensional residual strains in DKDP crystals with different deuterium contents are investigated by neutron diffraction technique. However, the pathway by which the defects affect the macroscopic stress in the grown crystal needs further research. The nonlinear absorption of 70% deuterated DKDP crystal at 355 nm has been assigned as two-photon absorption with the Z-scan method, which may contribute to the laser-induced damage.

For the preparation of DKDP crystals with excellent laser damage resistance, the purity of raw materials, continuous filtration technology, thermal annealing and laser conditioning are all key factors. Although the laser damage mechanism and laser conditioning mechanism are still not well understood, improvements of these processes enables the grown crystals to meet the specifications of the ICF laser system. Another remaining challenge is the lack of techniques to effectively characterize defects associated with the laser damage initiation. This makes it difficult to implement more efficient process improvements to further enhance the damage resistance of DKDP crystals. The simulation results of theoretical calculations can provide further insights into the experimental observations to a certain extent. Considering the difference brought about by isotopic effect, developing the pseudopotential of deuterium atom can make future theoretical calculations for DKDP crystals more accurate. In addition, as nonlinear optical crystals with outstanding performance, the applications of KDP/DKDP crystals and their analogs in fourth frequency generation, true zero-order waveplate, optical parametric chirped pulse amplification (OPCPA) still require further research.
